# No genetic causal association between COVID‐19 infection, hypogonadism, and male infertility

**DOI:** 10.1002/mco2.389

**Published:** 2023-09-24

**Authors:** Yang Xiong, Xiaokun Hu, Yangchang Zhang, Feng Qin, Jiuhong Yuan

**Affiliations:** ^1^ Department of Urology and Andrology Laboratory West China Hospital Sichuan University Chengdu Sichuan Province China; ^2^ Out‐patient Department, West China Hospital/West China School of Nursing Sichuan University Chengdu Sichuan Province China; ^3^ School of Public Health Capital Medical University Beijing China

Dear Editor:

SARS‐CoV‐2 infection has been observed to induce testicular damage, leading to a reduction in serum testosterone levels and sperm count.^1^ While the potential for hypogonadism and subfertility has been acknowledged, it remains inadequately assessed. Clinical studies present inconsistent findings regarding the relationship between COVID‐19 infection, hypogonadism, and male infertility.^2^ The true association between COVID‐19 infection, hypogonadism, and infertility remains unclear. To address the limitations of observational design, we employed the Mendelian Randomization (MR) approach in this study. MR is a method using single nucleotide polymorphisms (SNPs) as genetic proxies to substitute the exposures (i.e., COVID‐19 infection) and the outcomes (i.e., testosterone and male infertility).^3^ During gestation, SNPs closely associated with COVID‐19 infection, hypogonadism, and male infertility are distributed at random, which are not interfered by postnatal confounders.^3^ Therefore, within the framework of MR, patients with COVID‐19 infection are naturally randomized, facilitating the investigation of the risk of hypogonadism and infertility.

The Genome‐Wide Association Studies (GWASs) of COVID‐19 susceptibility (COVID vs. population), hospitalization (hospitalized COVID vs. population), and severity (very severe cases vs. population) were retrieved from the COVID‐19 Host Genetics Initiative.^4^ These GWASs included 1,683,768, 1,887,658, and 1,388,342 participants, respectively. The genetic associations of total testosterone (199,569 males), bioavailable testosterone (BAT, 184,205 males), and sex hormone‐binding globulin (SHBG, 185,221 males) and male infertility (680 cases and 72,799 controls) were extracted from previous GWASs and the FinnGen biobank.^5^ The detailed information of instrumental variables (IVs, *p* < 5 × 10^−8^) is displayed in Table S1. Further information regarding the GWASs and the downloading websites for the raw data can be found in Tables S2 and S3. *p* < 0.05/*n* (0.05/12 = 0.0042) was considered statistically significant. The statistical analyses are detailed in Supporting Information.

Our findings did not establish a causal link between COVID‐19 infection and male infertility. The Inverse Variance Weighted (IVW) method disclosed that the odds ratios (ORs) of male infertility were 0.74 for susceptibility (95% CI = 0.32–1.69, *p* = 0.472), 0.79 for hospitalization (95% CI = 0.53–1.19, *p* = 0.268), and 0.87 for severity (95% CI = 0.69–1.09, *p* = 0.220), respectively (Figure 1A). The other four methods including Maximum Likelihood, MR‐Egger, MR‐RAPS (Robust Adjusted Profile Score), and Weighted Median were in line with the IVW model, reporting no causal association between COVID‐19 infection and male infertility (all *p* > 0.05). Figure 1B–D illustrates an inverse correlation between the SNP effects on COVID‐19 infection (susceptibility, hospitalization, and severity) and male infertility. In Table S4, the intercept terms of MR‐Egger regression are close to zero (all *p* > 0.05), suggesting no pleiotropy. Additionally, the mean *F* statistics of selected IVs were greater than 48, indicating a lower likelihood of bias from weak IVs. In Figure S1, the funnel plots show symmetrical distributions of IVs. The IVW approach also reported no heterogeneity (all *p* > 0.05).

**FIGURE 1:**
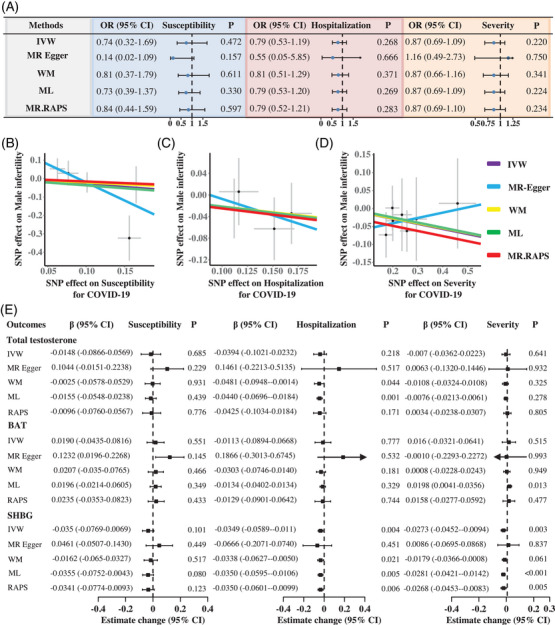
The causal association between COVID‐19 infection and male infertility. (A) Causal estimates between COVID‐19 susceptibility, hospitalization, severity, and male infertility. (B–D) The scatter plot visualizing the SNP effect of COVID‐19 susceptibility, hospitalization, and severity on male infertility. (E) Causal estimates between COVID‐19 infection and hypogonadism. COVID‐19: corona virus disease 2019; BAT: bioactive testosterone; SHBG: sex hormone‐binding globulin; MR: Mendelian Randomization; IVW: Inverse Variance Weighting; WM: Weighted Median; ML: Maximum Likelihood; MR‐RAPS: Robust Adjusted Profile Score.

In Figure 1E, the IVW method reveals that genetically proxied COVID‐19 infection has no impact on testosterone levels (*β* = 0.0148 for COVID‐19 susceptibility, 0.0394 for COVID‐19 hospitalization, and 0.0070 for COVID‐19 severity, all *p* > 0.05). The findings remained insignificant in most of the sensitivity analyses (all *p* > 0.05). The scatter plots did not exhibit a negative trend between the SNP effects on COVID‐19 infection and testosterone levels (Figure S2A–C). Of note, there was significant heterogeneity in the IVs (Figure S2D–F), without pleiotropy (Table S4 **)**.

Similarly, the IVW estimator found no significant associations between COVID‐19 infection and BAT by the IVW method in Figure 1E (*β* = 0.0190 for COVID‐19 susceptibility, −0.0113 for COVID‐19 hospitalization, and 0.0160 for COVID‐19 severity, all *p* > 0.05). The other four methods obtained similar results, providing no evidence that COVID‐19 infection leads to a decrease in BAT levels (all *p* > 0.05). In Figure S3A–C, no apparent downward trend is observed between the SNP effects on COVID‐19 infection and BAT. In Figure S3D–F and Table S4, significant heterogeneities are observed for the IVs of COVID‐19 hospitalization and severity (*p* < 0.05), without evidence of pleiotropy (*p* > 0.05).

No significant causal relationship was found between COVID‐19 susceptibility and SHBG levels (*β* = −0.0350, 95% CI = −0.0769 to 0.0069, *p* = 0.101; Figure 1E). However, the IVW estimator revealed that genetically predicted COVID‐19 hospitalization and severity were associated with decreased levels of SHBG (*β* = −0.0350, *p* = 0.004 and *β* = −0.0273, *p* = 0.003, respectively). The consistency in the magnitude and direction of results across MR‐RAPS, Weighted Median, and Maximum Likelihood methods substantiates the risk impact of COVID‐19 hospitalization and severity on SHBG levels. In Figure S4A–C, the scatter plots visualize a downward trend regarding the SNP effects between COVID‐19 infection and SHBG. In Figure S4D–F and Table S4, no significant heterogeneity and pleiotropy are detected (all *p* > 0.05).

We further performed reversed MR analyses to rule out reverse causality. In Figure S5, the IVW model shows that the increment of total testosterone, BAT, and SHBG concentrations have no effect on COVID‐19 infection (susceptibility, hospitalization, and severity, all *p* > 0.05). The mean *F* statistics were all greater than 80, showing adequate strengths of IVs. In addition, the intercept terms of MR‐Egger regression were close to null, indicating no pleiotropy (all *p* > 0.05). As shown in Figure S6, significant heterogeneities in the IVs for total testosterone, BAT, and SHBG are observed. Thus, the random‐effect IVW model was used to estimate the causal effects.

Notably, our study found a significant association between COVID‐19 infection and decreased SHBG levels. Previous reports have associated COVID‐19 infection with hepatic dysfunction.^6^ Given that SHBG is synthesized in the liver and transports sex hormones in serum, liver dysfunction may lead to decreased SHBG. A decline in SHBG indicates a higher concentration of free testosterone and a lower likelihood of hypogonadism and male infertility. In conclusion, our study provides causal evidence that genetically proxied COVID‐19 infection does not increase the risk of hypogonadism and male infertility.

## AUTHOR CONTRIBUTIONS

Jiuhong Yuan proposed the research topic and supervised the study. Yang Xiong, Xiaokun Hu, and Yangchang Zhang performed the statistical analysis. The manuscript was written by Yang Xiong and Xiaokun Hu, and revised by Jiuhong Yuan and Feng Qin. All authors have read and approved for the final manuscript.

## CONFLICT OF INTEREST STATEMENT

The authors declare they have no conflicts of interest.

## ETHICS STATEMENT

Ethical review and approval were waived for this study. The entire data from Mendelian Randomization is publicly accessible (https://gwas.mrcieu.ac.uk/). Informed consent was obtained from all subjects in the original genome‐wide association studies.

## Supporting information

Supporting Information

## Data Availability

The entire data from Mendelian Randomization is publicly accessible (https://gwas.mrcieu.ac.uk/).
