# Supplementary material for: No genetic causal association between COVID‐19 infection, hypogonadism, and male infertility
Source: MedComm (2020). 2023 Sep 24;4(5):e389. doi: 10.1002/mco2.389 (PMC10518432; doi:10.1002/mco2.389)
Supplement: Supplementary file 1 — Supporting Information [file MCO2-4-e389-s001.docx]

**Supplemental Information**

**No Genetic Causal Association between COVID-19 Infection, Hypogonadism and Male Infertility**

**Author information：**

Yang Xiong^a, #^, Xiaokun Hu^b,^ ^#^, Yangchang Zhang^c^, Feng Qin^a^, and Jiuhong Yuan^a,^ *

a. Department of Urology and Andrology Laboratory, West China Hospital, Sichuan University, Sichuan Province 610041, China;

b. Out-patient Department, West China Hospital/West China School of Nursing, Sichuan University, Chengdu, Sichuan Province, 610041, China;

c. School of Public Health, Capital Medical University, Beijing 100000, China.

# Yang Xiong and Xiaokun Hu contributed equally to this work.

*Correspondence should be addressed to: Prof. Jiuhong Yuan, Department of Urology and Andrology Laboratory, West China Hospital, Sichuan University, Sichuan Province 610041, China. Email: [jiuhongyuan2107@163.com](mailto:jiuhongyuan2107@163.com)

1. **Detailed Materials and Methods**

***1.1 Study population***

The Genome-wide association studies (GWASs) of COVID-19 susceptibility, hospitalization, and severity were retrieved from the COVID-19 Host Genetics Initiative [1]. Susceptibility to COVID-19 was defined as COVID-19 infection. Hospitalization to COVID-19 was defined as hospitalized COVID. Severity to COVID-19 was defined as very severe COVID cases. The controls were the general population without COVID-19 infection. The GWASs of COVID-19 susceptibility, hospitalization, and severity included 1,683,768 samples (38,984 cases and 1,644,784 controls), 1,887,658 samples (9,986 cases and 1,877,672 controls), and 1,388,342 samples (5,101 cases and 1,383,241 controls), respectively (detailed in **Table S2**). To avoid the population architecture bias, all the participants were from the European descent.

Summary-level statistics of total testosterone, bioavailable testosterone (BAT) and sex hormone binding globulin (SHBG) were extracted from previous GWASs [2]. Testosterone was detected using the competitive chemiluminescent enzyme immunoassay method and SHBG was determined using the sandwich immunoassay analysis on a Beckman Coulter Unicel Dxl 800. The concentration of BAT was estimated using the Vermeulen equation [3]. To avoid the bias from gender, we only extracted the male-specific genetic data to perform MR analyses. Participants who self-reported taking hormone-based medication were also excluded. Finally, the genetic associations of testosterone, BAT and SHBG were calculated based on 199,569, 184,205, and 185,221 males with European descent. Age and ten principal components were adjusted in the linear mixed models. The effects of identified variants were scaled to one SD increase of androgen. The heritability of testosterone, BAT and SHBG was 17%, 12% and 21%, respectively.

The genetic association of male infertility was obtained from FinnGen biobank (<https://r5.finngen.fi/>). In FinnGen, male sterility referred to permanent infertility and was defined as the inability of the male to effect fertilization of an ovum after a specified period of unprotected intercourse. The diagnosis of male infertility was according to the codes of international classification of diseases (N46 for ICD-10; 606 for ICD-8 and ICD-9). To reduce bias, participants with male reproductive organ cancer such as the penis and scrotum were excluded in the included samples. Finally, 680 cases and 72,799 controls were included in the GWAS.

***1.2 Statistical analyses***

All the genetic associations were obtained from the European descent at the genome-wide significance threshold (*P* < 5 ×10^-8^), subsequently clumped at a 10000Kb window with *r*^2^ < 0.01. SNPs with minor effect allele were excluded since they were rare variants in the population. Additionally, MR-Steiger filtering method was used to exclude reverse causality. To evaluate the strength the SNPs, F-statistics were calculated using the following formula: F-statistics = (Beta/Se)^2^. Generally, the average F statistics < 10 indicated the presence of weak instrumental variable bias, which should be excluded from the MR analyses. In this study, there were no signs of weak instrumental variable bias (all F statistics > 10, **Table S1**).

The inverse variance weighting (IVW) method was considered as the main analysis and used to combine the Wald ratio of SNPs. In addition, the MR-Egger, weighted median, maximum likelihood, and robust adjusted profile score (RAPS) approaches were used as supplements for sensitivity analyses. To exclude reverse causality, we further performed reverse MR, which used testosterone, BAT, and SHBG as exposures and the COVID-19 infection as outcomes. The criteria for selecting IVs were the same as those stated in the previous procedures. The insignificant results indicated that hypogonadism could not reversely lead to COVID-19 infection, providing more reliability to our results.

The Cochrane’Q test was adopted to quantify the heterogeneity, and MR-Egger intercept method was used for detecting horizontal pleiotropy. Detailed information of instrumental variables (IVs) is displayed in **Table S1**. *P* < 0.05 (two sided) was suggestively significant in statistics. To control for the type I error rate, the Bonferroni correction was used to adjust for multiple testing. Thus, *P* < 0.05/n (0.05/12 = 0.0042) was considered statistically significant. MR analyses were performed using the R 4.0.2 software (R Foundation for Statistical Computing, Vienna, Austria), with the “TwoSampleMR”, and “mr.raps” package.

**References:**

[1] COVID-19 Host Genetics Initiative. The COVID-19 Host Genetics Initiative, a global initiative to elucidate the role of host genetic factors in susceptibility and severity of the SARS-CoV-2 virus pandemic. *Eur J Hum Genet*. 2020;28(6):715-718.

[2] Ruth KS, Day FR, Tyrrell J, et al. Using human genetics to understand the disease impacts of testosterone in men and women. *Nat Med.* 2020;26(2):252-258.

[3] Chung MC, Gombar S, Shi RZ. Implementation of Automated Calculation of Free and Bioavailable Testosterone in Epic Beaker Laboratory Information System. *J Pathol Inform*. 2017; 8:28.

1. **Table S1: Instrumental variables for susceptibility, hospitalization, and severity of COVID-19 in male infertility.**

| **Exposures** | **Outcomes** | **SNP** | **Chr** | **Position** | **A1** | **A2** | **EAF** | **Beta** | **SE** | **P values** |
| --- | --- | --- | --- | --- | --- | --- | --- | --- | --- | --- |
| **Susceptibility** | **Male infertility** | rs10936744 | 3 | 101433440 | T | C | 0.3588 | -0.0626 | 0.0100 | 3.51E-10 |
|  |  | rs2271616 | 3 | 45838013 | T | G | 0.1181 | 0.1563 | 0.0151 | 3.61E-25 |
|  |  | rs35508621 | 3 | 45880481 | C | T | 0.0834 | 0.1652 | 0.0171 | 4.72E-22 |
|  |  | rs4971066 | 1 | 155105882 | G | T | 0.1777 | -0.0768 | 0.0134 | 1.02E-08 |
|  |  | rs643434 | 9 | 136142355 | A | G | 0.3710 | 0.1013 | 0.0101 | 1.29E-23 |
|  | **Total testosterone** | rs10936744 | 3 | 101433440 | T | C | 0.3588 | -0.0626 | 0.0100 | 3.51E-10 |
|  |  | rs2271616 | 3 | 45838013 | T | G | 0.1181 | 0.1563 | 0.0151 | 3.61E-25 |
|  |  | rs35508621 | 3 | 45880481 | C | T | 0.0834 | 0.1652 | 0.0171 | 4.72E-22 |
|  |  | rs4971066 | 1 | 155105882 | G | T | 0.1777 | -0.0768 | 0.0134 | 1.02E-08 |
|  | **BAT** | rs10936744 | 3 | 101433440 | T | C | 0.3588 | -0.0626 | 0.0100 | 3.51E-10 |
|  |  | rs2271616 | 3 | 45838013 | T | G | 0.1181 | 0.1563 | 0.0151 | 3.61E-25 |
|  |  | rs35508621 | 3 | 45880481 | C | T | 0.0834 | 0.1652 | 0.0171 | 4.72E-22 |
|  |  | rs4971066 | 1 | 155105882 | G | T | 0.1777 | -0.0768 | 0.0134 | 1.02E-08 |
|  | **SHBG** | rs10936744 | 3 | 101433440 | T | C | 0.3588 | -0.0626 | 0.0100 | 3.51E-10 |
|  |  | rs2271616 | 3 | 45838013 | T | G | 0.1181 | 0.1563 | 0.0151 | 3.61E-25 |
|  |  | rs35508621 | 3 | 45880481 | C | T | 0.0834 | 0.1652 | 0.0171 | 4.72E-22 |
|  |  | rs4971066 | 1 | 155105882 | G | T | 0.1777 | -0.0768 | 0.0134 | 1.02E-08 |
| **Hospitalization** | **Male infertility** | rs13050728 | 21 | 34615210 | C | T | 0.6528 | -0.1683 | 0.0202 | 7.44E-17 |
|  |  | rs2109069 | 19 | 4719443 | A | G | 0.3227 | 0.1513 | 0.0199 | 2.94E-14 |
|  |  | rs2660 | 12 | 113357442 | A | G | 0.6902 | 0.1164 | 0.0194 | 2.00E-09 |
|  |  | rs505922 | 9 | 136149229 | C | T | 0.3501 | 0.1118 | 0.0191 | 4.42E-09 |
|  | **Total testosterone** | rs13050728 | 21 | 34615210 | C | T | 0.6528 | -0.1683 | 0.0202 | 7.44E-17 |
|  |  | rs2109069 | 19 | 4719443 | A | G | 0.3227 | 0.1513 | 0.0199 | 2.94E-14 |
|  |  | rs2660 | 12 | 113357442 | A | G | 0.6902 | 0.1164 | 0.0194 | 2.00E-09 |
|  |  | rs505922 | 9 | 136149229 | C | T | 0.3501 | 0.1118 | 0.0191 | 4.42E-09 |
|  | **BAT** | rs13050728 | 21 | 34615210 | C | T | 0.6528 | -0.1683 | 0.0202 | 7.44E-17 |
|  |  | rs2109069 | 19 | 4719443 | A | G | 0.3227 | 0.1513 | 0.0199 | 2.94E-14 |
|  |  | rs2660 | 12 | 113357442 | A | G | 0.6902 | 0.1164 | 0.0194 | 2.00E-09 |
|  |  | rs505922 | 9 | 136149229 | C | T | 0.3501 | 0.1118 | 0.0191 | 4.42E-09 |
|  | **SHBG** | rs13050728 | 21 | 34615210 | C | T | 0.6528 | -0.1683 | 0.0202 | 7.44E-17 |
|  |  | rs2109069 | 19 | 4719443 | A | G | 0.3227 | 0.1513 | 0.0199 | 2.94E-14 |
|  |  | rs2660 | 12 | 113357442 | A | G | 0.6902 | 0.1164 | 0.0194 | 2.00E-09 |
|  |  | rs505922 | 9 | 136149229 | C | T | 0.3501 | 0.1118 | 0.0191 | 4.42E-09 |
| **Severity** | **Male infertility** | rs11085727 | 19 | 10466123 | T | C | 0.2811 | 0.1727 | 0.0293 | 3.74E-09 |
|  |  | rs111837807 | 6 | 31121232 | C | T | 0.0996 | 0.2945 | 0.0428 | 5.66E-12 |
|  |  | rs13050728 | 21 | 34615210 | C | T | 0.6627 | -0.2001 | 0.0286 | 2.44E-12 |
|  |  | rs2109069 | 19 | 4719443 | A | G | 0.3287 | 0.2566 | 0.0281 | 6.12E-20 |
|  |  | rs2237698 | 7 | 107607902 | T | C | 0.0897 | 0.2366 | 0.0397 | 2.41E-09 |
|  |  | rs2384074 | 12 | 113382977 | T | C | 0.6756 | 0.1982 | 0.0282 | 2.10E-12 |
|  |  | rs77534576 | 17 | 47940666 | T | C | 0.0347 | 0.4598 | 0.0749 | 8.52E-10 |
|  | **Total testosterone** | rs11085727 | 19 | 10466123 | T | C | 0.2811 | 0.1727 | 0.0293 | 3.74E-09 |
|  |  | rs111837807 | 6 | 31121232 | C | T | 0.0996 | 0.2945 | 0.0428 | 5.66E-12 |
|  |  | rs13050728 | 21 | 34615210 | C | T | 0.6627 | -0.2001 | 0.0286 | 2.44E-12 |
|  |  | rs2109069 | 19 | 4719443 | A | G | 0.3287 | 0.2566 | 0.0281 | 6.12E-20 |
|  |  | rs2237698 | 7 | 107607902 | T | C | 0.0897 | 0.2366 | 0.0397 | 2.41E-09 |
|  |  | rs2384074 | 12 | 113382977 | T | C | 0.6756 | 0.1982 | 0.0282 | 2.10E-12 |
|  |  | rs77534576 | 17 | 47940666 | T | C | 0.0347 | 0.4598 | 0.0749 | 8.52E-10 |
|  | **BAT** | rs11085727 | 19 | 10466123 | T | C | 0.2811 | 0.1727 | 0.0293 | 3.74E-09 |
|  |  | rs111837807 | 6 | 31121232 | C | T | 0.0996 | 0.2945 | 0.0428 | 5.66E-12 |
|  |  | rs13050728 | 21 | 34615210 | C | T | 0.6627 | -0.2001 | 0.0286 | 2.44E-12 |
|  |  | rs2109069 | 19 | 4719443 | A | G | 0.3287 | 0.2566 | 0.0281 | 6.12E-20 |
|  |  | rs2237698 | 7 | 107607902 | T | C | 0.0897 | 0.2366 | 0.0397 | 2.41E-09 |
|  |  | rs2384074 | 12 | 113382977 | T | C | 0.6756 | 0.1982 | 0.0282 | 2.10E-12 |
|  |  | rs77534576 | 17 | 47940666 | T | C | 0.0347 | 0.4598 | 0.0749 | 8.52E-10 |
|  | **SHBG** | rs11085727 | 19 | 10466123 | T | C | 0.2811 | 0.1727 | 0.0293 | 3.74E-09 |
|  |  | rs111837807 | 6 | 31121232 | C | T | 0.0996 | 0.2945 | 0.0428 | 5.66E-12 |
|  |  | rs13050728 | 21 | 34615210 | C | T | 0.6627 | -0.2001 | 0.0286 | 2.44E-12 |
|  |  | rs2109069 | 19 | 4719443 | A | G | 0.3287 | 0.2566 | 0.0281 | 6.12E-20 |
|  |  | rs2237698 | 7 | 107607902 | T | C | 0.0897 | 0.2366 | 0.0397 | 2.41E-09 |
|  |  | rs2384074 | 12 | 113382977 | T | C | 0.6756 | 0.1982 | 0.0282 | 2.10E-12 |
|  |  | rs77534576 | 17 | 47940666 | T | C | 0.0347 | 0.4598 | 0.0749 | 8.52E-10 |

Notes: BAT: bioavailable testosterone; SHBG: sex hormone binding globulin; SNP: single nucleotide polymorphisms; EAF: effect allele frequency; SE: standard error.

**3. Table S2: Detailed information of included GWASs.**

| **Traits** | **Sample sizes** | **Race** | **PMID** |
| --- | --- | --- | --- |
| COVID-19 susceptibility | 1,683,768 samples (38,984 cases and 1,644,784 controls) | European descent | 32404885 |
| COVID-19 hospitalization | 1,887,658 samples (9,986 cases and 1,877,672 controls) | European descent | 32404885 |
| COVID-19 severity | 1,388,342 samples (5,101 cases and 1,383,241 controls) | European descent | 32404885 |
| Male infertility | 73,479 samples (680 cases, and 72,799 controls) | European descent | - |
| Total testosterone | 199,569 males | European descent | 32042192 |
| BAT | 184,205 males | European descent | 32042192 |
| SHBG | 185,221 males | European descent | 32042192 |

Notes: BAT: bioavailable testosterone; SHBG: sex hormone binding globulin; GWAS: Genome-wide association studies; PMID: PubMed Identifier.

| **4. Table S3: Data sources and downloading websites.** | | | |
| --- | --- | --- | --- |
| **Traits** | **GWAS ID** | **Download website** | **Dataset size** |
| Testosterone | ieu-b-4865 | https://gwas.mrcieu.ac.uk/datasets/ieu-b-4865/ | 367 MB |
| BAT | ieu-b-4868 | https://gwas.mrcieu.ac.uk/datasets/ieu-b-4868/ | 367 MB |
| SHBG | ieu-b-4871 | https://gwas.mrcieu.ac.uk/datasets/ieu-b-4871/ | 367 MB |
| Male infertility | finn-b-N14_MALEINFERT | https://storage.googleapis.com/finngen-public-data-r5/  summary_stats/finngen_R5_N14_MALEINFERT.gz | 536 MB |
| COVID-19 susceptibility | ebi-a-GCST011073 | Visiting through API:  https://gwas.mrcieu.ac.uk/datasets/ebi-a-GCST011073/  or  Raw data: https://www.covid19hg.org/results/ | 225 MB |
| COVID-19 hospitalization | ebi-a-GCST011081 | Visiting through API:  https://gwas.mrcieu.ac.uk/datasets/ebi-a-GCST011081/  or  Raw data: https://www.covid19hg.org/results/ | 300 MB |
| COVID-19 severity | ebi-a-GCST011075 | Visiting through API:  https://gwas.mrcieu.ac.uk/datasets/ebi-a-GCST011075/  or  Raw data: https://www.covid19hg.org/results/ | 298 MB |

**5. Table S4: Heterogeneity and pleiotropy of used instrumental variables.**

| **Exposures** | **Outcomes** | **Number of SNPs** | **F-statistics** | **Intercept for Egger regression** | **P for Egger** |
| --- | --- | --- | --- | --- | --- |
| **Susceptibility** | **Male infertility** | 5 | 74.63 | 0.1763 | 0.191 |
|  | **Total testosterone** | 4 | 68.20 | -0.0140 | 0.166 |
|  | **BAT** | 4 | 68.20 | -0.0122 | 0.164 |
|  | **SHBG** | 4 | 68.20 | -0.0095 | 0.214 |
| **Hospitalization** | **Male infertility** | 4 | 49.43 | 0.0532 | 0.782 |
|  | **Total testosterone** | 4 | 49.43 | -0.0261 | 0.421 |
|  | **BAT** | 4 | 49.43 | -0.0278 | 0.505 |
|  | **SHBG** | 4 | 49.43 | 0.0044 | 0.698 |
| **Severity** | **Male infertility** | 7 | 48.22 | -0.0705 | 0.524 |
|  | **Total testosterone** | 7 | 48.22 | -0.0031 | 0.854 |
|  | **BAT** | 7 | 48.22 | 0.0040 | 0.887 |
|  | **SHBG** | 7 | 48.22 | -0.0084 | 0.397 |

Notes: BAT: bioavailable testosterone; SHBG: sex hormone binding globulin; SNP: single nucleotide polymorphisms.

**6. Figure S1: Heterogeneities of IVs in detecting the association between COVID-19 infection and male infertility.**


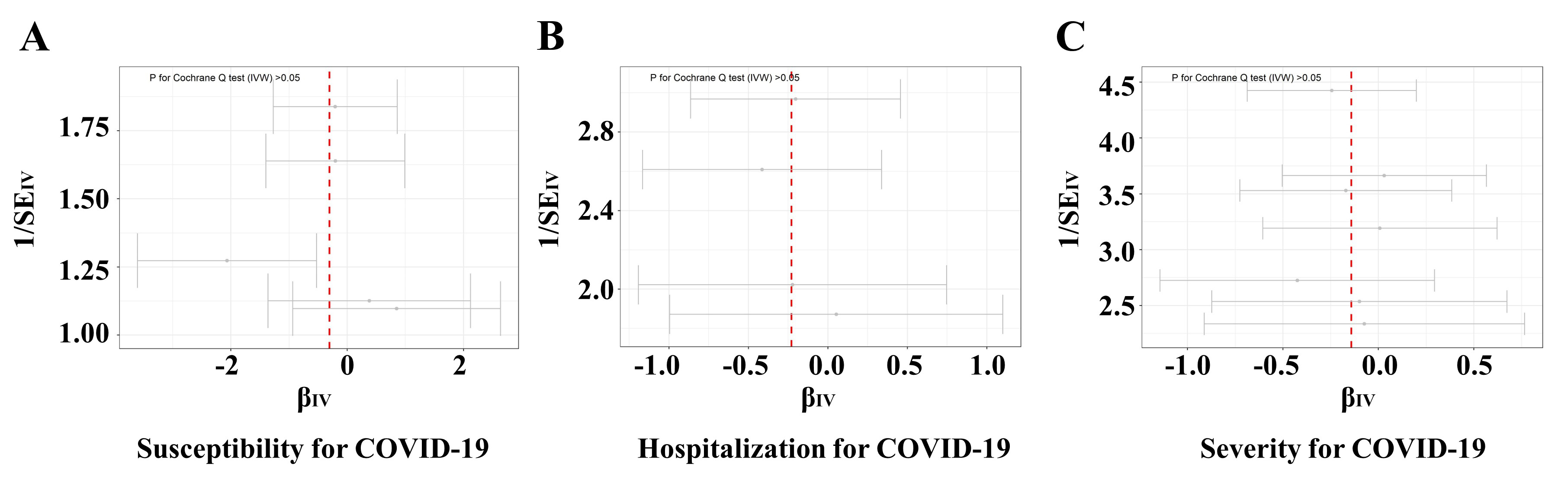


Notes: The heterogeneities of COVID-19 susceptibility, hospitalization, and severity were visualized in Figure S1A, S1B, and S1C, respectively. The outcome was Male infertility.

**7. Figure S2: Scatter plots and Funnel plots visualizing the SNP effects and heterogeneities of IVs in detecting the association between COVID-19 infection and total testosterone.**


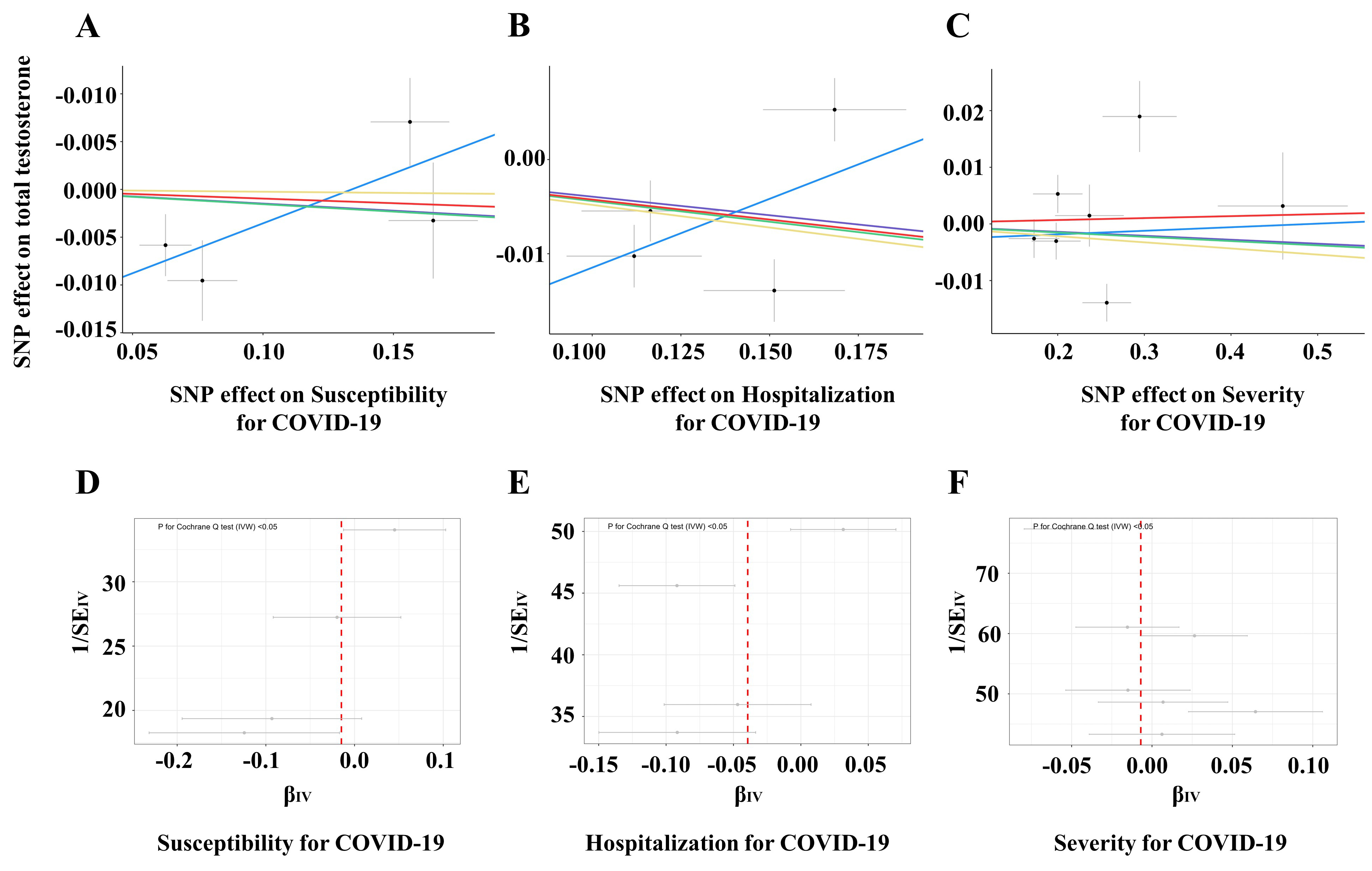


Notes: The SNP effects of COVID-19 susceptibility, hospitalization, and severity on total testosterone were visualized in Figure S2A, S2B, and S2C, respectively. The heterogeneities of COVID-19 susceptibility, hospitalization, and severity were visualized in Figure S2D, S2E, and S2F, respectively. The outcome was total testosterone.

**8. Figure S3: Scatter plots and Funnel plots visualizing the SNP effects and heterogeneities of IVs in detecting the association between COVID-19 infection and BAT.**


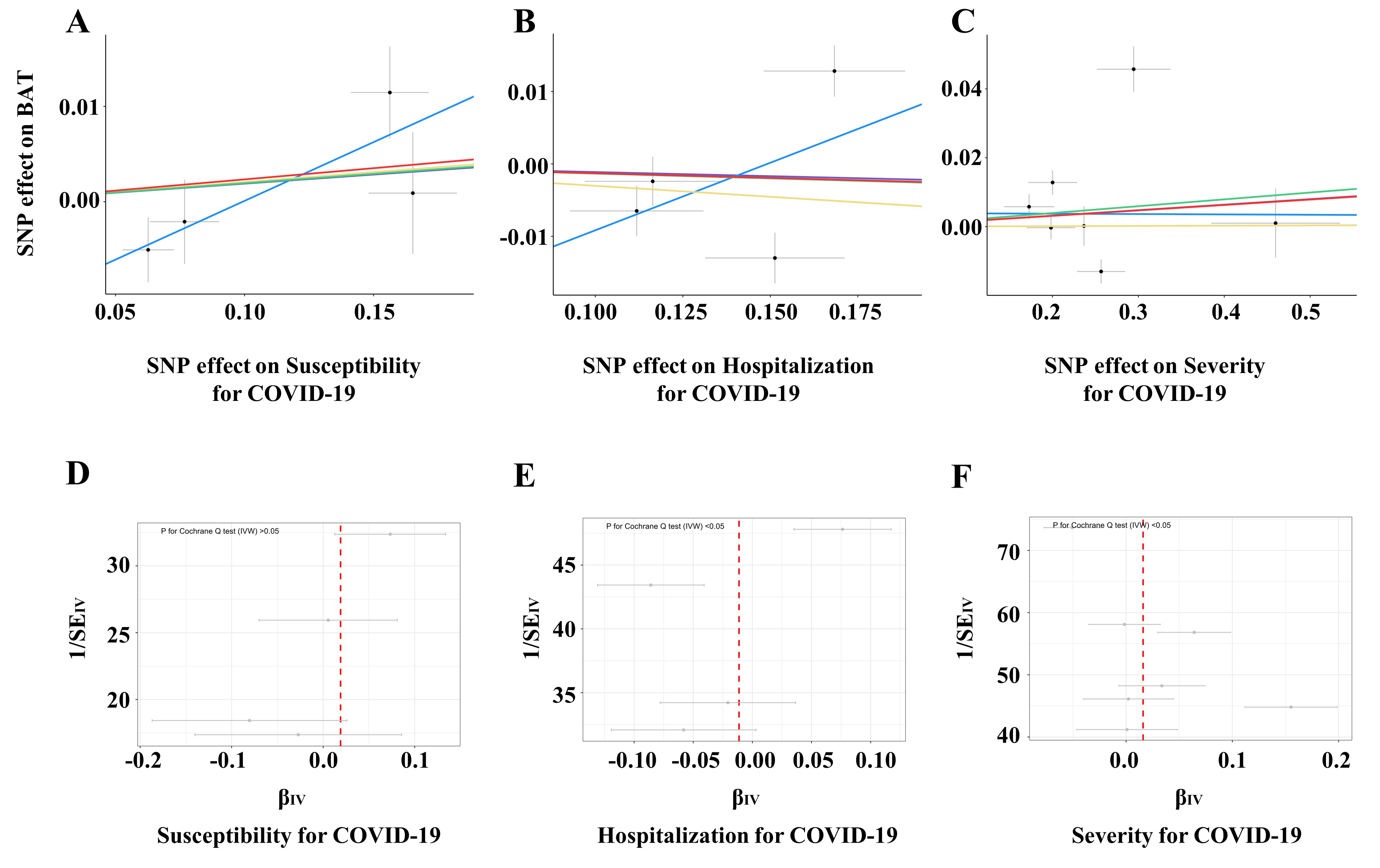


Notes: The SNP effects of COVID-19 susceptibility, hospitalization, and severity on total testosterone were visualized in Figure S3A, S3B, and S3C, respectively. The heterogeneities of COVID-19 susceptibility, hospitalization, and severity were visualized in Figure S3D, S3E, and S3F, respectively. The outcome was BAT. BAT: bioavailable testosterone.

**9. Figure S4: Scatter plots and Funnel plots visualizing the SNP effects and heterogeneities of IVs in detecting the association between COVID-19 infection and SHBG.**


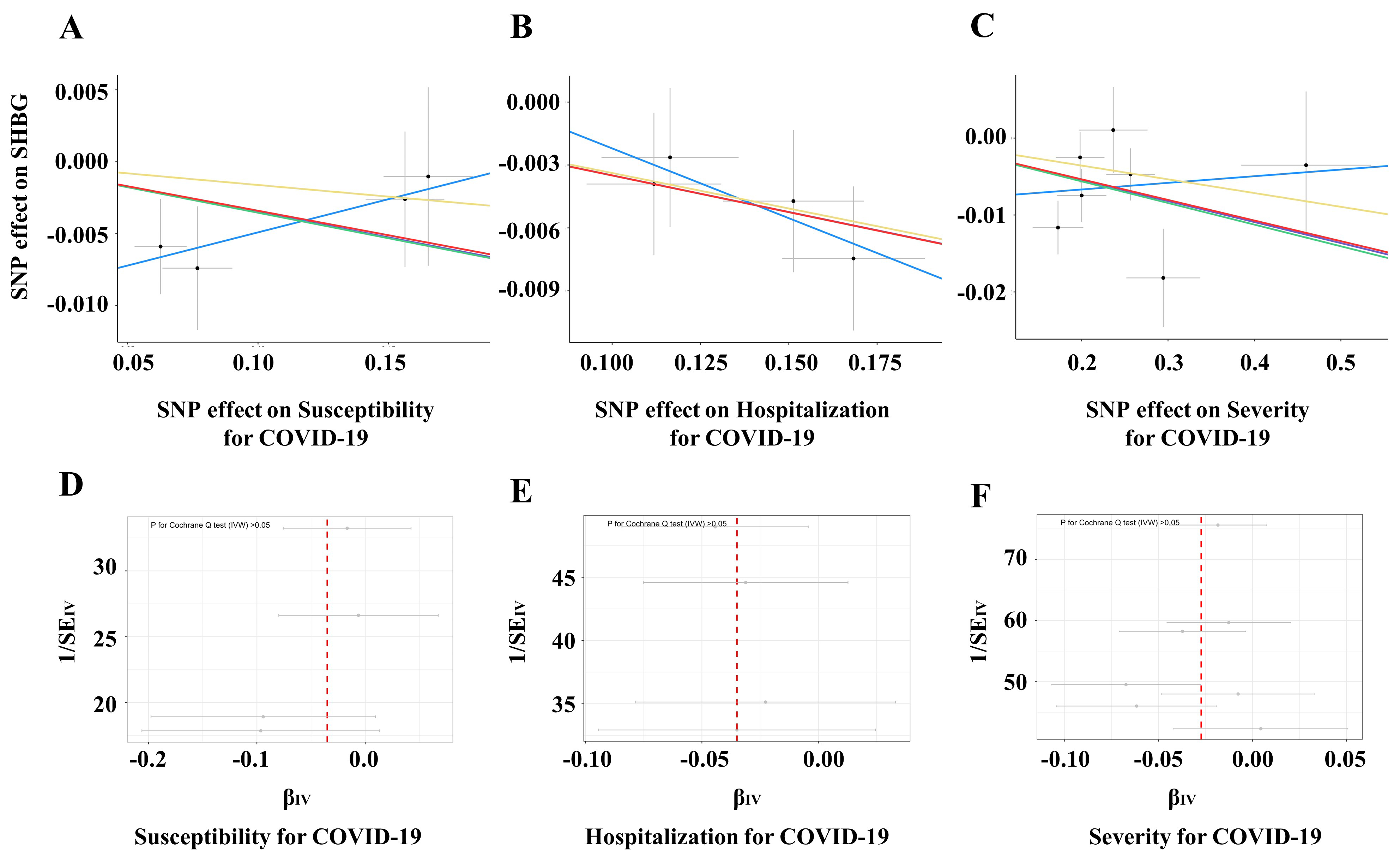


Notes: The SNP effects of COVID-19 susceptibility, hospitalization, and severity on total testosterone were visualized in Figure S4A, S4B, and S4C, respectively. The heterogeneities of COVID-19 susceptibility, hospitalization, and severity were visualized in Figure S4D, S4E, and S4F, respectively. The outcome was SHBG. SHBG: sex hormone binding globulin.

**10. Figure S5: Results of reversed Mendelian Randomization analyses.**


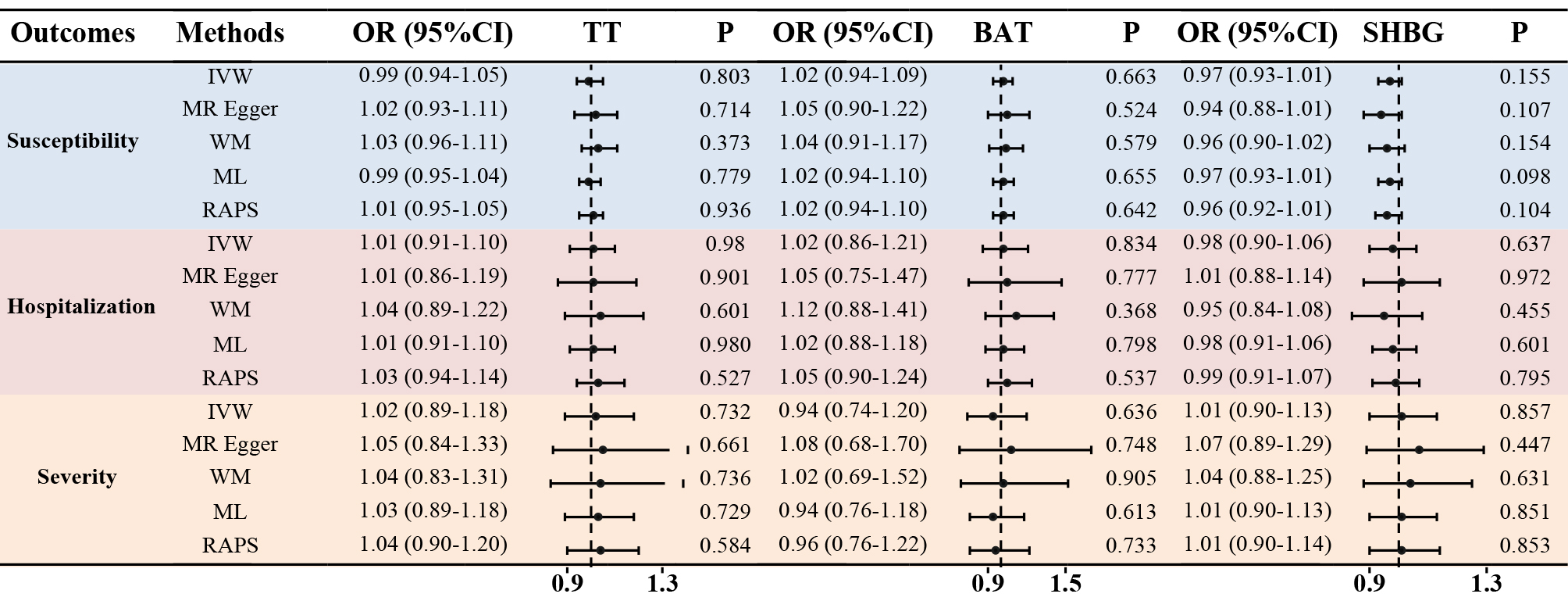


Notes: OR: odds ratio; CI: confidence interval; TT: total testosterone; BAT: bioavailable testosterone. SHBG: sex hormone binding globulin; WM: weighted median; ML: maximum likelihood.

**11. Figure S6: Heterogeneities of IVs in the reverse Mendelian Randomization analysis.**


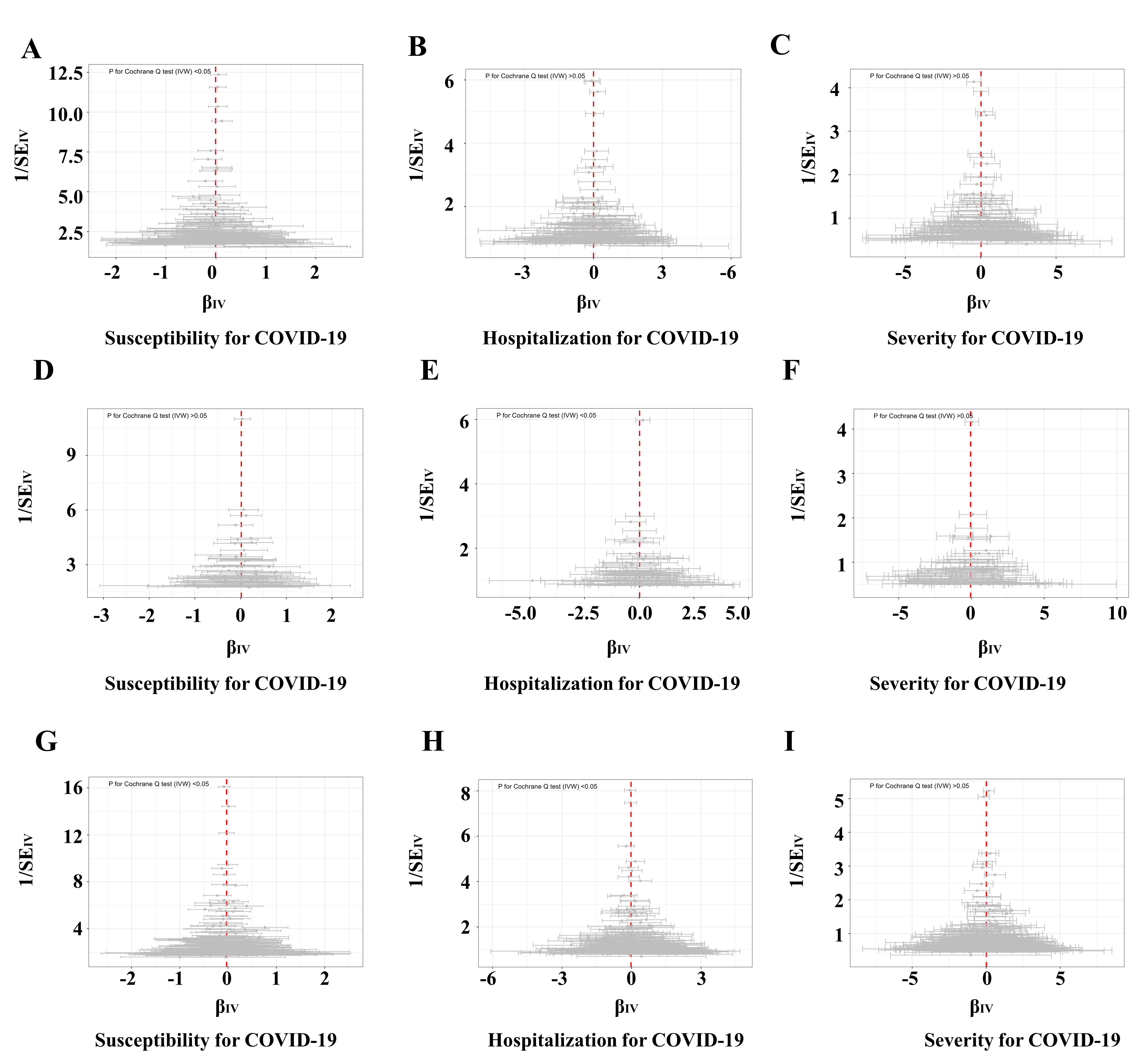


Notes: The heterogeneities of instrumental variables in detecting the association between total testosterone and susceptibility, hospitalization, and severity to COVID-19 were visualized in Figure S6A, S6B, and S6C, respectively. Similarly, Figure S6D, S6E, and S6F visualized the heterogeneities of instrumental variables for BAT. Figure S6G, S6H, and S6I visualized the heterogeneities of instrumental variables for SHBG.

**Author contributions**

Jiuhong Yuan proposed the research topic and supervised the study. Yang Xiong, Xiaokun Hu, and Yangchang Zhang performed the statistical analysis. The manuscript was written by Yang Xiong and Xiaokun Hu and revised by Jiuhong Yuan, and Feng Qin.

**Acknowledgments**

We thank Ms. Xiaoyingzi Huang of the Andrology Lab, West China hospital, Sichuan University, for technical assistance.

**Conflicts of interest statement**

The authors declare no conflicts of interest.

**Funding information**

This work was supported by the Natural Science Foundation of China (81871147 & 82071639).

**Data availability statement**

All the data from Mendelian randomization is publicly accessible (<https://gwas.mrcieu.ac.uk/>).

**Ethics statement**

Ethical review and approval were waived for this study, all the data from Mendelian randomization is publicly accessible (https://gwas.mrcieu.ac.uk/). Informed consent was obtained from all subjects in the original genome-wide association studies.
